# Fine-needle aspiration of the thyroid: an overview

**DOI:** 10.1186/1742-6413-2-12

**Published:** 2005-06-29

**Authors:** Gia-Khanh Nguyen, Mark W Lee, Jody Ginsberg, Tina Wragg, Darcy Bilodeau

**Affiliations:** 1 Department of Laboratory Medicine and Pathology, University of Alberta Hospital, Edmonton, Alberta, Canada; 2 Department of Medicine (Endocrinology and Metabolism), University of Alberta Hospital, Edmonton, Alberta, Canada

## Abstract

Thyroid nodules (TN) are a common clinical problem. Fine needle aspiration (FNA) of the thyroid now is practiced worldwide and proves to be the most economical and reliable diagnostic procedure to identify TNs that need surgical excision and TNs that can be managed conservatively. The key for the success of thyroid FNA consists of an adequate or representative cell sample and the expertise in thyroid cytology. The FNA cytologic manifestations of TNs may be classified into seven working cytodiagnostic groups consisting of a few heterogenous lesions each to facilitate the differential diagnosis. Recent application of diagnostic molecular techniques to aspirated thyroid cells proved to be useful in separating benign from malignant TNs in several cases of indeterminate lesions.

## 

Fine-needle aspiration (FNA) for cytologic evaluation of thyroid cancer was originally used by Martin and Ellis at New York Memorial Hospital for Cancer and Allied Diseases in 1930 [1]. However, this diagnostic procedure was subsequently found to have a limited value, and it was then discontinued at the above-mentioned institution [2,3]. The thyroid FNA was not further developed and did not gain acceptance in the United States for nearly 50 years until the early 1980s when its diagnostic value was firmly demonstrated by Scandinavian investigators [4-8]. The 1974 report by Crockford and Bain [9] and the 1979 paper of Miller and Hamburger [10] were apparently the first North American publications attesting to the value of thyroid FNA. This method of clinical investigation now is practiced worldwide and has become the cornerstone in the management of thyroid nodules (TN) [11-25].

## Indication and Goal of Thyroid FNA

Thyroid nodular lesions are a common clinical problem. In the United States, 4 to 7% of the adult population have a palpable TN [13]. The incidence of thyroid cancer in a clinically solitary TN or in a multinodular goiter is equal and about 5% in non-endemic areas [26]. TNs constitute the main indication for FNA, and the goal of this diagnostic procedure is to detect thyroid neoplasms for surgical resection and to identify non-neoplastic lesions that may be managed conservatively [23]. This method of clinical investigation has reduced the number of diagnostic thyroid surgeries for TNs by 60–85%, and the difference in rates of thyroid surgery reflect the cytodiagnostic accuracy rates among different medical centers [24].

## Contraindications and Complications of Thyroid FNA

The main contraindication to thyroid FNA is bleeding diathesis, as the formation of a large hematoma at the biopsy site may cause compression of the trachea and respiratory distress [13,23]. Therefore, a bleeding time, PT and PTT should be ordered to screen this condition in all patients prior to thyroid FNA. This diagnostic procedure, if properly performed, is almost free of complications. Subcutaneous hematoma at the biopsy site, accidental puncture of the trachea and local infection are rare complications [13]. Hematoma may be prevented by local pressure of the overlying skin at the biopsy site [13]. Tracheal injury is manifested by minimal and transient hemoptysis. Seeding of thyroid cancer cells along the needle tract is also an exceedingly rare complication with FNA [13].

## Procuration and Preparation of Cell Samples

### 1. Procurement of cell samples

Obtaining an adequate or satisfactory cell sample for cytologic evaluation is not simple, and interpreting thyroid cytology is challenging and requires expertise [13,23]. To perform thyroid FNA, the TN is identified by palpation, and a 22- to 25-gauge and 4.5-cm-long needle is commonly used to procure cell samples from at least three different areas of any TN. Usually, only dermal anesthesia is required. Depending on personal preferences FNA of a TN may be performed either with or without a syringe [13]. However, for cystic thyroid lesions, the cyst contents should be evacuated first by FNA with a syringe. The gland is then carefully examined by palpation. If a residual nodule is found, it should be aspirated. If the TN is difficult to identify by palpation the patient should be referred to a radiologist for FNA under ultrasonographic guidance [13,22-24]. Since the thyroid is rich in capillary blood vessels the needle aspirate usually contains a large amount of peripheral blood that may be reduced by limiting the biopsy procedure to about five seconds or by using the FNA technique without aspiration [13].

### 2. Preparation of cell samples

For cytological evaluation, smears should be appropriately prepared and stained. Depending on the amount and nature of the thyroid needle aspirates one of the following preparation techniques is used: (a). A small drop of thyroid aspirate is put near the frosted end of a glass slide and is quickly and gently smeared by a cover slip. (b). A small drop of thyroid aspirate is put on a glass slide and gently crushed with a second slide that is then separated vertically from the first one. (c). A small or medium-sized drop of thyroid aspirate is put near the frosted end of a slide that is placed on a table. A second slide is used to spread the aspirated material in the same manner used to prepare a peripheral blood smear. (d) Cytospin smears should be prepared from the liquid contents of all cystic thyroid lesions. (e). Excess of aspirated material should be used for preparation of a cell block that may show diagnostic tissue fragments on sectioning. It is important that a small drop of aspirated material is used for smear preparation, as if a large drop of aspirate material is used, an unevenly thick smear may be obtained, and at the end of the slide a thick and bloody cell film may be formed. This will obscure the cellular details of underlying thyroid cells and tissue fragments, making their evaluation extremely difficult, if not impossible.

### 3. Routine staining methods

Depending on personal preference, either air-dried and Romanowsky-stained smears or ethanol-fixed and Papanicolaou-stained smears are prepared. For Papanicolaou staining, the smears must be fixed quickly before drying with 95% ethanol or with a commercial spray fixative. A delay in fixation will result in air-dried artefactual changes with loss of cellular details. Air-dried smears for staining with one of the Romanowsky modified methods (Wright stain, May-Grunwald-Giemsa or Diff-Quik method) now are widely used, as air-drying artifactual changes can be avoided. However, nuclear details in Romanowsky-stained smears are not as well-visualized as in wet-fixed and Papanicolaou-stained smears. A parallel use of air-dried and wet-fixed smears is usually recommended, as these two staining methods are complementary [13,22,23]. Fixation of aspiration smears in Carnoy solution for 3–5 minutes may be used to lyse red blood cells prior to staining with the Papanicolaou method.

## Specimen Adequacy

Obtaining an adequate cell sample is a prerequisite to the success of thyroid cytology. Therefore, immediate microscopic assessment of the needle aspirate by a pathologist or a cytotechnologist is desirable. If the first sample is judged inadequate for cytological evaluation, the TN can be re-aspirated immediately. If a rapid evaluation is not available, multiple FNAs of different areas of the TN should be performed.

The range of inadequate or unsatisfactory specimens reported in the literature ranges from 2–21% (means 17%) [15]. Currently, criteria for specimen adequacy vary from institution to institution. Some investigators require that an adequate sample should contains five to six groups of well-preserved and well-visualized follicular cells with each group containing 10 or more cells [12]. One group requires multiple punctures of the TN to be evaluated, with at least six properly prepared smears and a minimum of 8–10 tissue fragments of well-preserved follicular epithelium on each of two slides [25]. Another group requires 10 clusters of follicular cells with at least 20 cells in each cluster [13]. The Papanicolaou Society of Cytopathology Task Forces on Standard of Practice does not specify any numbers and groups of thyroid follicular epithelial cells for specimen adequacy [23]. Two practical exceptions to these adequacy criteria are applied: (a) a benign colloid nodule may be suggested if a large amount of thick colloid material is present, regardless of the number of follicular epithelial cell clusters [23]; or, (b) if a cell sample contains one or two small clusters of malignant or highly atypical cells, it should be reported as malignant or suspicious for malignancy and not as unsatisfactory or inadequate for cytodiagnosis [23]. Thyroid FNA under ultrasonographic guidance achieved higher rates of adequate cell samples, in the range of 79–99.3% (mean, 91%) [21,27-36]. Ultrasound-guided thyroid FNA proved to be useful in sampling TNs smaller than 2 cm in greatest dimension, complex or solid-cystic TNs [27-36] and abnormal thyroid beds [35,36].

## Cytodiagnosis and Its Limitations

The cytodiagnosis of TNs by FNA is complex for the following reasons [26]:

a. overlap of cytological patterns between neoplastic and non-neoplastic lesions.

b. overlap of cytological features between various neoplasms.

c. coexistence of non-neoplastic and neoplastic processes and multiple malignancies in the same gland.

For a practical diagnostic approach, the cytological findings of thyroid lesions may be divided into seven main groups, as recommended by the Papanicolaou Task Force on Standard of Practice [23]. These groups are heterogenous and consist of both neoplastic and non-neoplastic lesions that may show either similar or specific cytological manifestations [23]. A non-diagnostic group is added as some TNs yield inadequate or non-specific cytological findings. The above-mentioned groups with their commonly encountered thyroid lesions are tabulated in Table 1.

**Table 1:** Cytodiagnostic Groups with Commonly Encountered Thyroid Nodular Lesions*.

1. Benign colloid nodule	- Solitary colloid nodule - Prominent nodule in MNG - Macrofollicular adenoma
2. Cellular microfollicular lesion	- Microfollicular adenoma - Low-grade follicular carcinoma - Hyperplastic microfollicular lesions in HT or MNG

3. Hurthle cell lesion	- Hurthle cell adenoma - Hurthle cell carcinoma - Hyperplastic Hurthle cell nodule in HT or MNG

4. Primary malignant tumor	- Papillary carcinoma - High-grade microfollicular carcinoma - Insular carcinoma - Medullary carcinoma - Anaplastic carcinoma - Lymphoma

5. Cystic lesions	- Benign colloid nodule - Papillary carcinoma - Other thyroid neoplasms

6. Thyroiditis	- Acute thyroiditis - Hashimoto thyroiditis - Subacute thyroiditis

7. Other lesions	- Graves disease - Metastatic cancer

8. Non-diagnostic category	-

* HT, Hashimoto thyroiditis; MNG, multinodular colloid goiter

### 1. Benign Colloid Nodule

**Figure 1:**
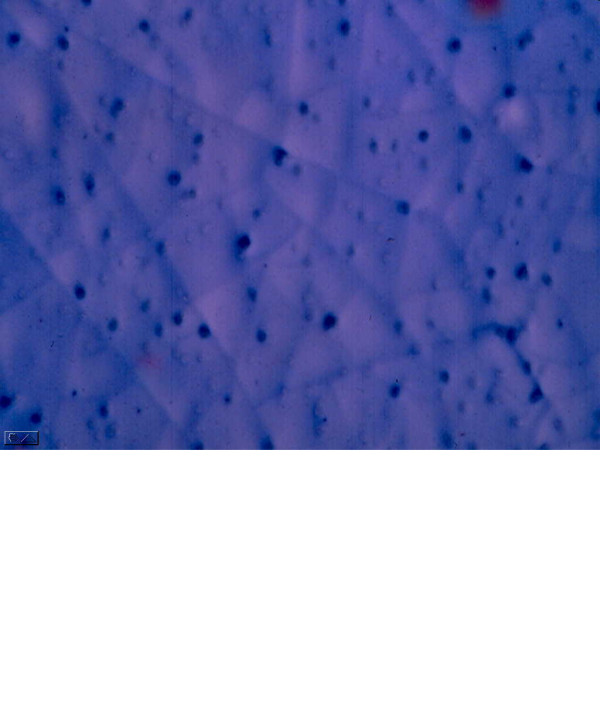
Thick, deep blue colloid material with cracking pattern in FNA of a benign colloid nodule (Diff-Quik stain, × 250).

**Figure 2:**
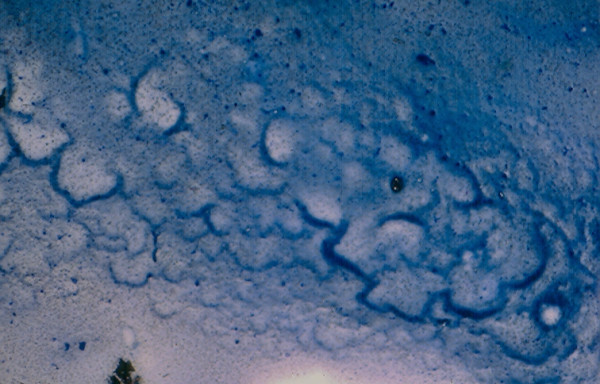
Thick, deep blue colloid material with bubble pattern in FNA of a benign colloid nodule (Diff-Quik stain, × 250) view).

**Figure 3:**
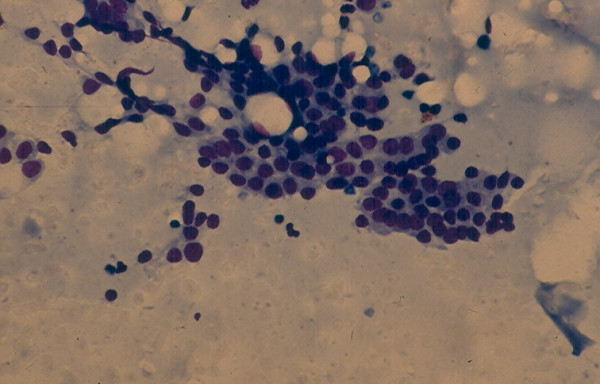
A monolayered sheet of benign follicular epithelial cells with honeycomb pattern in FNA of a benign colloid nodule (Diff-Quik stain, × 400).

This group includes *solitary benign colloid nodules *and prominent *benign colloid nodules in a multinodular colloid goiter*. These lesions arecharacterized by abundant, thick colloid material with cracking or bubble pattern (Figs. 1 and 2) and sheets of benign follicular epithelial cells in honeycomb arrangement (Fig. 3). Clusters of slightly hyperplastic Hurthle cells may be present [12,22,23,25]. The cytological differential diagnosis between a benign colloid nodule and a *macrofollicular adenoma *of the thyroid is extremely difficult if not impossible, as the two lesions usually show abundant, thick colloid and similar follicular cells [22,23].

### 2. Cellular Microfollicular Lesion

**Figure 4:**
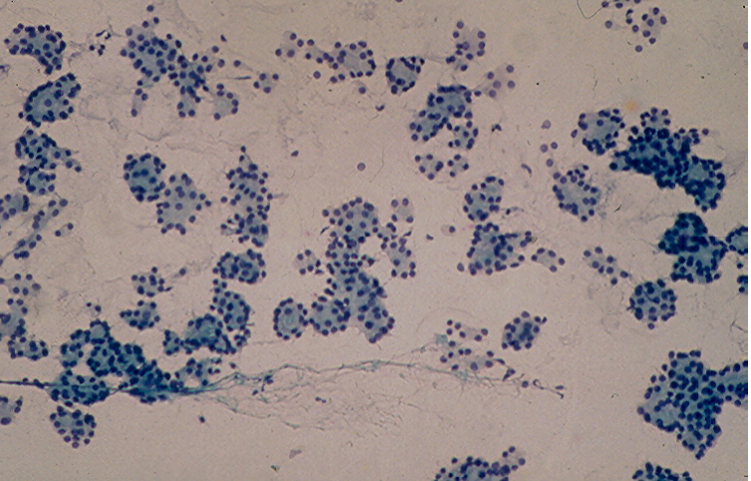
Cellular microfollicular lesion showing in FNA cells with round nuclei arranged in acini and small monolayered sheet (Papanicolaou stain, 4 × 160 and 5 × 400).

**Figure 5:**
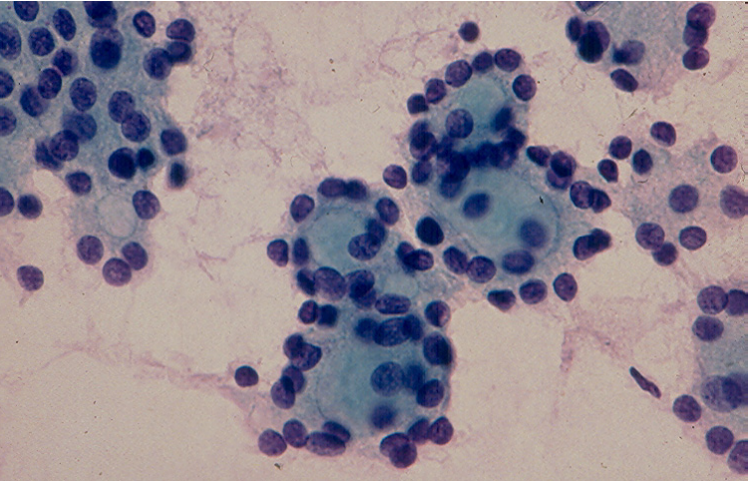
Cellular microfollicular lesion showing in FNA cells with round nuclei arranged in acini (Papanicolaou stain, 4 × 160 and 5 × 400).

This group includes *hyperplastic microfollicular nodules *in a multinodular colloid goiter or Hashimoto thyroiditis, a *microfollicular adenoma*, and a *well-differentiated follicular carcinoma*. These lesions are the most challenging ones to diagnose cytologically [22-25]. They are commonly reported as a microfollicular lesion or tumor with a recommendation for surgical excision [13,22-24]. FNA from a microfollicular lesion usually reveals abundant follicular cells in clusters, acini and small monolayered sheets (Figs 4 and 5). The individual cells show scanty, ill-defined cytoplasm and oval nuclei with regular nuclear contours and inconspicuous or prominent nucleoli.

Cellular microfollicular lesions of the thyroid fall into the diagnostic category of indeterminate or suspicious lesions [14,15,22], and in one large series 14% of microfollicular lesions were malignant [12].

### 3. Hurthle Cell Lesion

**Figure 6:**
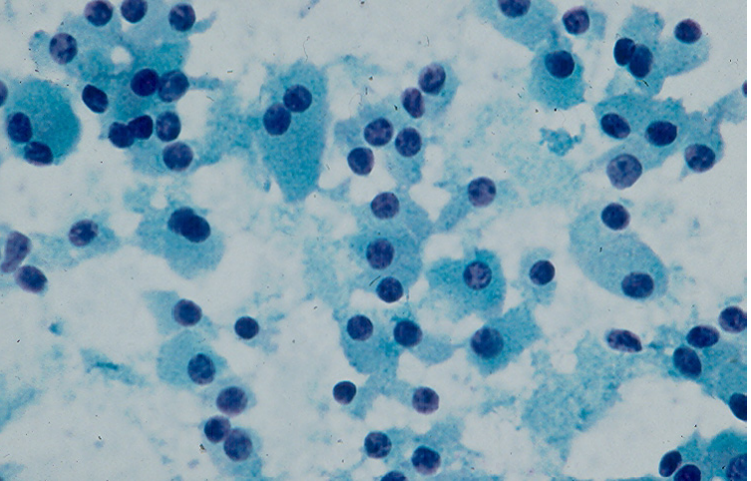
Hurthle cells with abundant, granular cytoplasm and round, central or eccentrically located nuclei in FNA of a Hurthle cell lesion (Papanicolaou stain, × 400).

**Figure 7:**
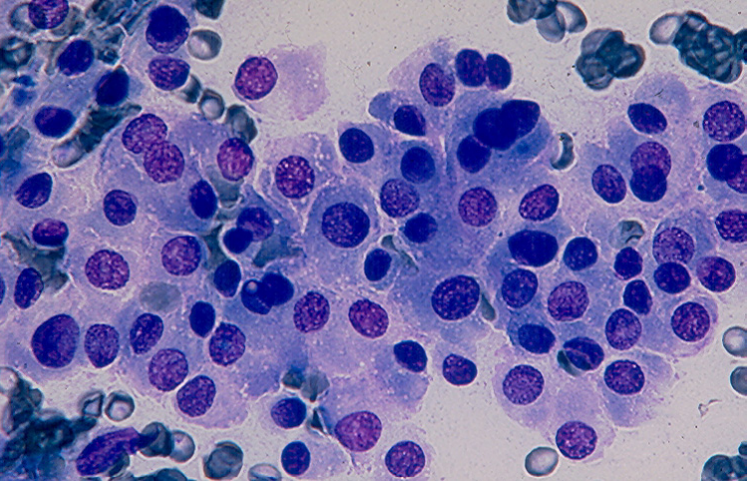
Hurthle cells in loose, monolayered sheet and singly in FNA of a Hurthle cell lesion (Diff-Quik stain, × 400).

Diagnosis of Hurthle cell lesions is a challenge in thyroid cytology. A *hyperplastic Hurthle cell nodule *in a Hashimoto thyroiditis or in a multinodular colloid goiter and a *Hurthle cell neoplasm *display similar cytologic findings [22-25,37,38]. The presence of numerous lymphocytes or a large amount of thick colloid material in the needle aspirate may indicate a hyperplastic Hurthle cell nodule in Hashimoto disease or a multinodular colloid goiter, respectively [38]. Hurthle cell adenoma and carcinoma usually show similar cytologic findings that are characterized by sheets and clusters of polygonal epithelial cells with abundant, granular, eosinophilic or basophilic cytoplasm, oval nuclei with regular nuclear contours and conspicuous or inconspicuous nucleoli [22-25] (Figs. 6 and 7). The presence of syncytial clusters of Hurthle cells with or without prominent nuclei [25] and abundant naked tumor cell nuclei has been reported to be a feature of Hurthle cell carcinoma [38].

When a Hurthle cell lesion is detected by FNA, surgical excision is usually indicated for further histologic study [38]. Thyroid Hurthle cell lesions fall into the cytodiagnostic category of indeterminate lesions or suspected malignant lesions [14,15,22], and 13% of Hurthle cell lesions were malignant in one large series [12].

### 4. Primary Malignant Lesions

This group includes *papillary, high-grade follicular, insular, medullary *and *anaplastic carcinomas*, and *lymphoma*. These lesions commonly show distinctive cytologic features that permit a correct identification in the majority of cases [13,24,25]. An *insular carcinoma*, or poorly differentiated carcinoma yields small cells in clusters, similar to those of a high-grade microfollicular carcinoma [39].

**Figure 8:**
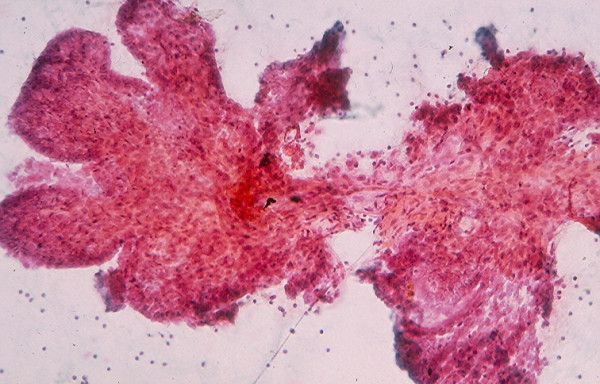
Thick branching papillary tissue fragment with fibrovascular core in FNA of a conventional papillary carcinoma (Papanicolaou stain, × 100).

**Figure 9:**
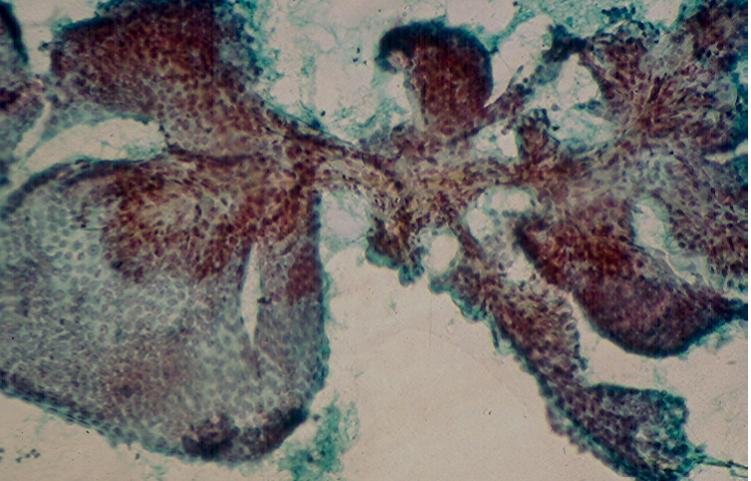
Thin branching papillary tissue fragment with fibrovascular core in FNA of a conventional papillary carcinoma (Papanicolaou stain, × 100).

**Figure 10:**
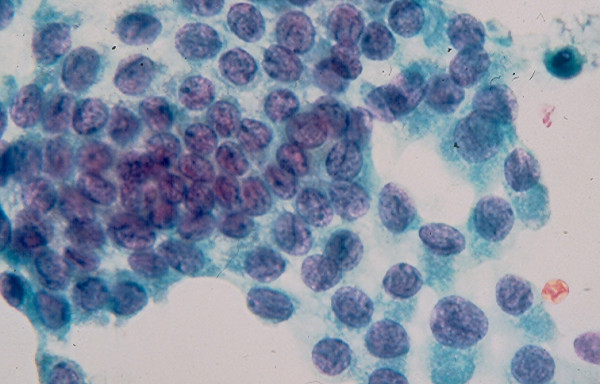
A sheet of tumor cells showing focal nuclear crowding with several cells displaying nuclear grooves in FNA of a conventional papillary carcinoma (Papanicolaou stain, × 400).

**Figure 11:**
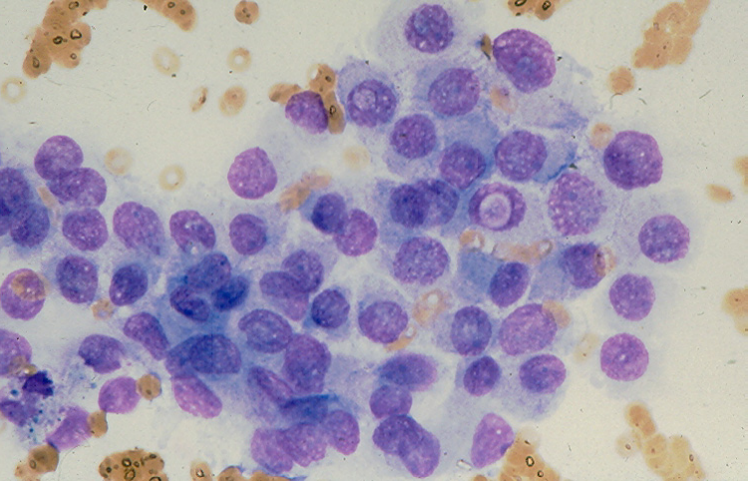
A loose sheet of tumor cells showing minimal nuclear crowding and two cells with intranuclear cytoplasmic inclusions in FNA of a conventional papillary carcinoma (Diff-Quik stain, × 400).

**Figure 12:**
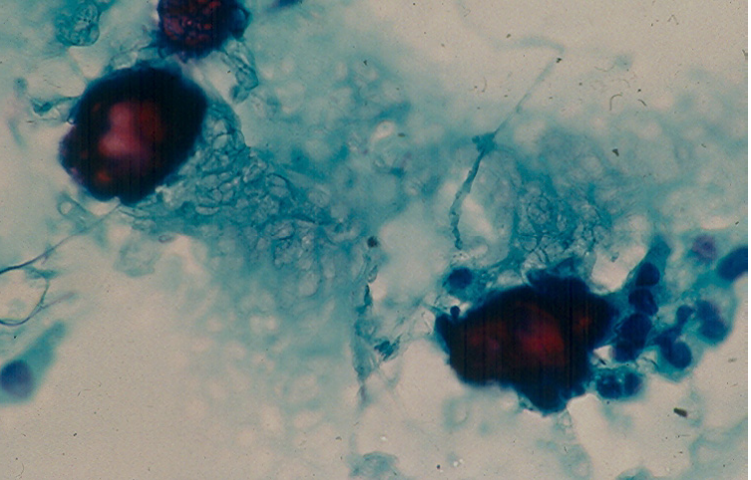
Two psammoma bodies in a smear showing a small amount of colloid material. A small aggregate of poorly preserved follicular cells is seen beside one psammoma body (Papanicolaou stain, × 400).

**Figure 13:**
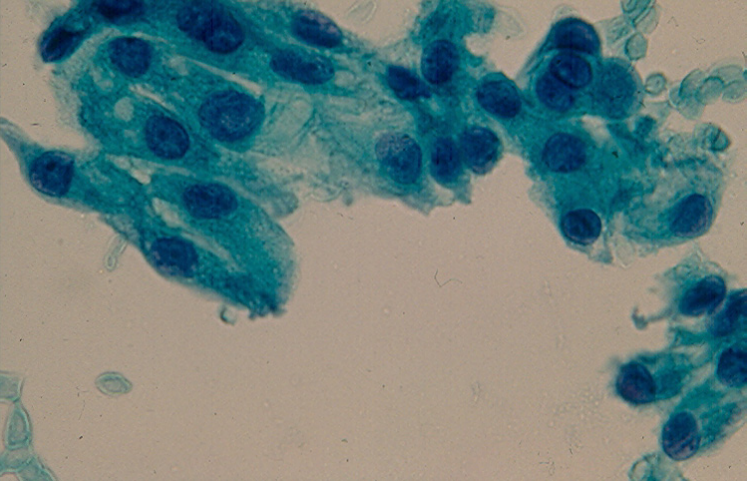
A loose cluster of metaplastic squamous cells seen in FNA of a conventional papillary carcinoma (Papanicolaou stain, × 400).

4a. *Papillary carcinoma *(PC). The conventional PC is characterized in FNA by the presence of thick or thin papillary tissue fragments with fibrovascular cores, sheets of tumor cells showing focal nuclear crowding and overlapping, irregular nuclear contours, intranuclear cytoplasmic inclusions (INCI) and nuclear grooves (NG). Psammoma bodies and metaplastic squamous cells may also be present [13,22,24,25] (Figs. 8, 9, 10, 11, 12, 13). These nuclear changes are recognized with less difficulty in Papanicolaou-stained cell samples, but they may be difficult to identify in cell samples stained with the Romanowsky staining method [13,22,23]. However, a presence of minute true papillary tissue fragments with fibrous vascular cores even without the identifiable above-mentioned nuclear changes is indicative of a PC. These papillary tissue fragments should be differentiated from thick and large follicular epithelial cell clusters with vascular transgression that may be found in FNA from different types of non-papillary epithelial neoplasms of the gland [40].

**Figure 14:**
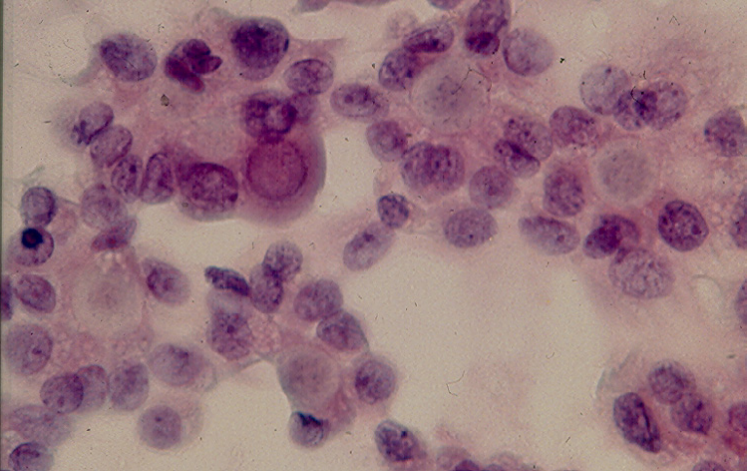
Papillary carcinoma, microfollicular variant showing in FNA cells in acinar arrangement. A tumor cell with an intranuclear cytoplasmic inclusion is noted (Papanicolaou stain, × 400).

*- Micro- *and *macrofollicular PCs *constitute a diagnostic challenge. A microfollicular PC may show in FNA follicular cells forming acini similar to those seen in the aforementioned cellular microfollicular lesions, and a macrofollicular PC may be easily mistaken for a macrofollicular adenoma or a benign colloid nodule cytologically, as nuclear changes characteristic for a thyroid PC may not be seen [41-43] (Fig. 14).

*- Hyalinizing trabecular adenoma *is indistinguishable from a PC cytologically, as these two lesions yield cells with similar nuclear features [44]. Recent molecular studies have suggested that this tumor is actually an encapsulated *trabecular variant of thyroid PC *[45].

**Figure 15:**
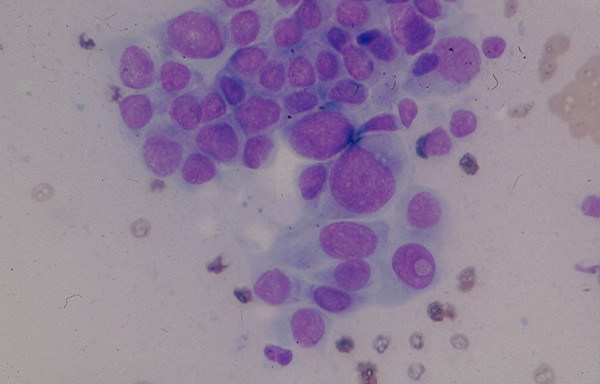
Papillary carcinoma, tall-cell variant showing in FNA a sheet of pleomorphic cells with some cells with elongated configuration and cytoplasmic tails. A tumor cell with intranuclear cytoplasmic inclusion is present (Diff-Quik stain, × 400).

**Figure 16:**
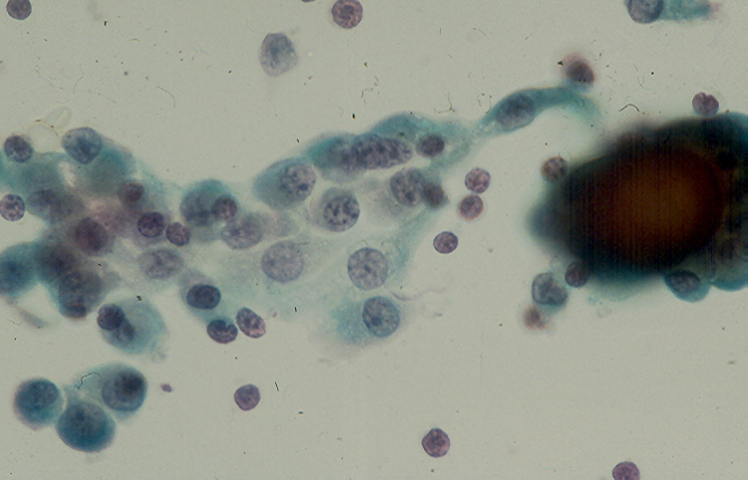
Papillary carcinoma, diffuse sclerosing type showing in FNA a sheet of metaplastic squamous cells, scattered lymphocytes and a psammoma body (Papanicolaou stain, × 400).

*- Other PC carcinoma subtypes*. *Tall-cell PC *is characterized by the presence of tall tumor cells with well-defined, granular cytoplasm and nuclei with NGs and single or multiple INCIs, making at least 30% of the aspirated cells [46-51] (Fig. 15). *Columnar-cell variant *shows no classic cytologic features of thyroid PC, but presence of clusters of columnar cells with palisading nuclei and the absence of classic nuclear changes of thyroid PC are cellular features of this neoplasm [52]. *Diffuse sclerosing variant *can be confidently suggested when abundant squamous cells admixed with lymphocytes, follicular epithelial cells with nuclear features of papillary carcinoma and a few psammoma bodies are noted [53,54] (Fig. 16).

4b. A *high-grade follicular carcinoma *and *insular carcinoma *are characterized by sheets and acinar clusters of pleomorphic epithelial cells with prominent nucleoli [22,24,25].

**Figure 17:**
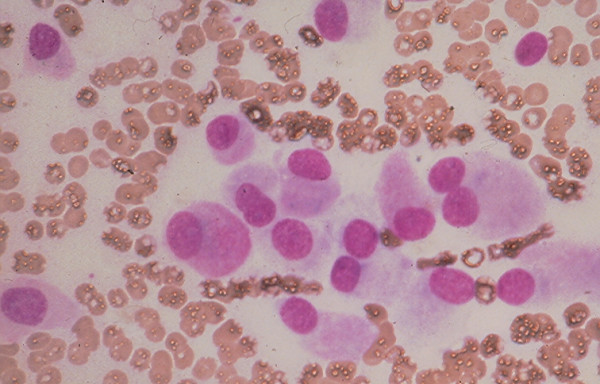
Medullary carcinoma showing in FNA dyshesive plasmacytoid tumor cells with eccentrically located round nuclei and intracytoplasmic azurophil granules (Diff-Quik stain, × 400).

**Figure 18:**
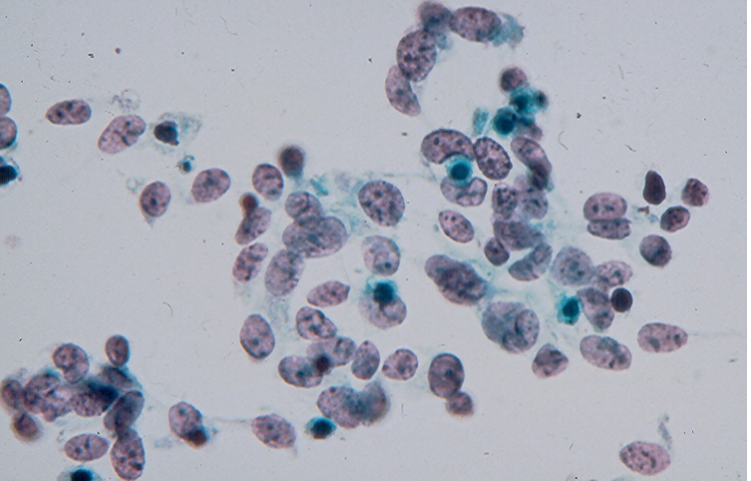
Medullary carcinoma showing in FNA loosely clustered spindle-shaped tumor cells with scanty, ill-defined cytoplasm (Papanicolaou stain, × 400).

**Figure 19:**
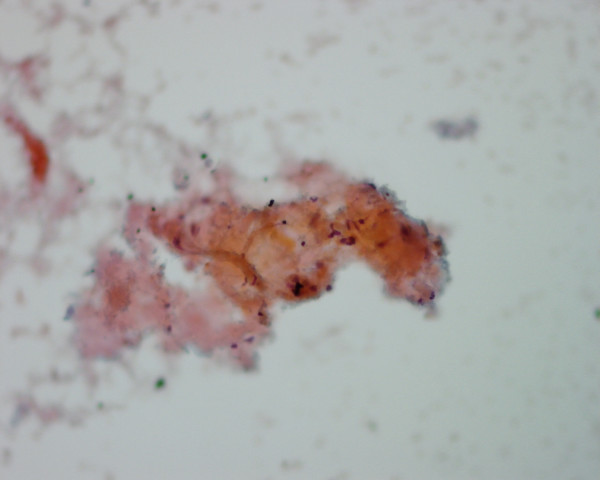
A fragment of orange and granular amyloid material seen in FNA of a thyroid medullary carcinoma (Papanicolaou stain, × 400).

4c. A *medullary carcinoma *shows in FNA a mixture of single and clustered polygonal cells and spindle tumor cells that may display INCIs [22,24,25] (Figs. 17 and 18). The tumor cells cytoplasm may show intracytoplasmic pink azurophil granules that are well-visualized by MGG or Diff-Quik stain and stain positively with calcitonin antibody. Amyloid material that stains positively with Congo red may be seen (Fig. 19).

**Figure 20:**
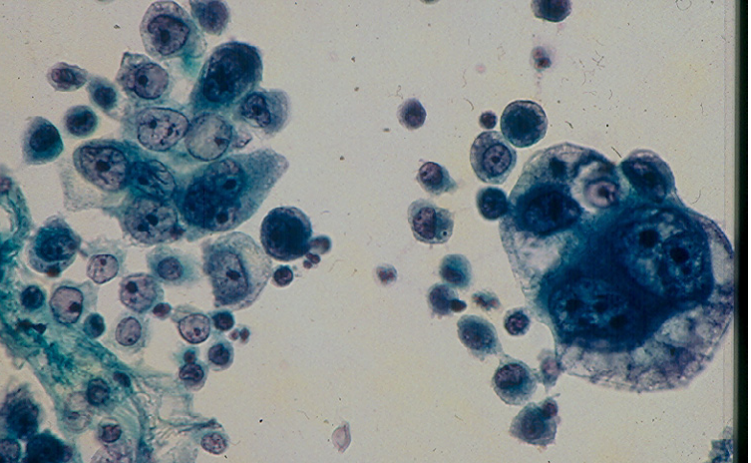
Anaplastic carcinoma, giant-cell type showing in FNA single and clustered large, bizarre malignant cells with pleomorphic nuclei and prominent nucleoli (Papanicolaou stain, × 400).

**Figure 21:**
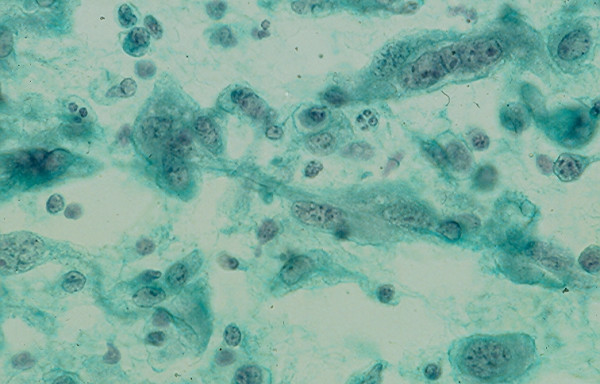
Anaplastic carcinoma, spindle-cell type showing in FNA dyshesive spindle- shaped malignant cells with scant, ill-defined cytoplasm (Papanicolaou stain, × 400).

4.d. A *naplastic thyroid carcinoma consists of two main histologic variants: Giant cell and spindle cell-subtypes*. Depending on the histologic subtype, an anaplastic thyroid carcinoma may display in FNA pleomorphic large, bizarre cancer cells with prominent nucleoli or spindle cancer cells admixed with a variable amount of necrotic debris (Figs. 20 and 21).

4.e. A primary *thyroid non-Hodgkin lymphoma *is usually of large cell type and yields in FNA cells similar to those of a lymph node involved by the same neoplastic process. A *thyroid Hodgkin disease *is characterized by Reed-Sternberg cells admixed with benign lymphoid cells and eosinophils [13,24,25].

### 5. Cystic Lesion

**Figure 22:**
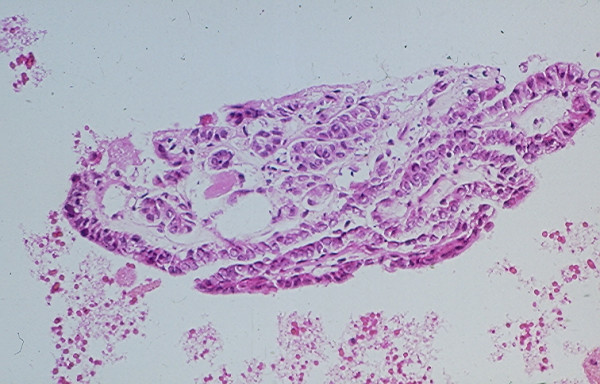
Papillary tissue fragments with thin fibrovascular cores covered with epithelial cells displaying nuclear crowding and occasional intranuclear cytoplasmic inclusions seen in a cell block section prepared from the needle aspirate of a papillary carcinoma with hemorrhagic cystic degenerative change (hematoxylin and eosin stain, × 250).

**Figure 23:**
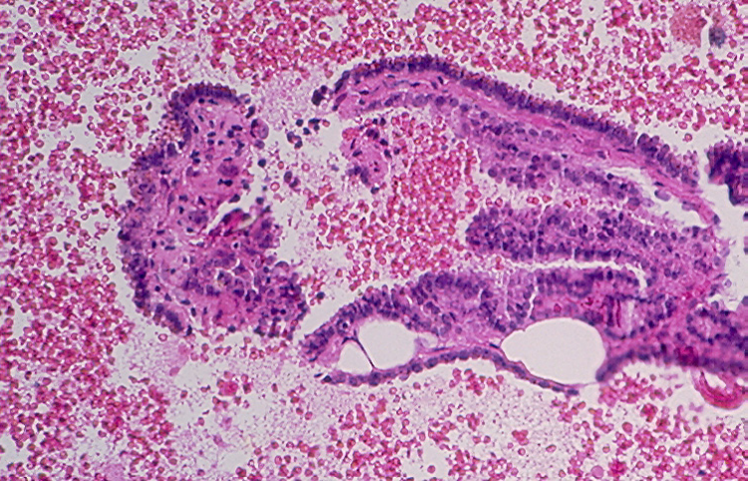
Section from a cell block prepared from the needle aspirate of a benign colloid nodule with hemorrhagic cystic degenerative change showing papillary tissue fragments covered with epithelium displaying no nuclear changes characteristic for a papillary carcinoma (hematoxylin and eosin stain, × 250).

Benign cysts account for the majority of thyroid cystic lesions. They are formed as the result of hemorrhagic degeneration of a benign colloid nodule. FNA from a benign colloid cyst may show colloid material admixed with benign follicular epithelial cells and hemosiderin laden macrophages. However, any thyroid neoplasm may undergo hemorrhagic necrosis and become a cystic lesion [13,22-25]. Of the thyroid neoplasms, PC tends to undergo marked hemorrhagic degenerative change. FNA from the tumor commonly shows a large amount of blood and the cystic lesion tends to recur rapidly [23]. Cytological examination of the aspiration smears usually reveals a large amount of blood and rarely tumor cells. However, sections from the cell block prepared from the needle aspirate may show diagnostic papillary tissue fragments with fibrovascular cores or nuclear features of a PC [23] while that of a benign colloid nodule will show no true papillary tissue fragments with fibrovascular cores or nuclear features of a thyroid PC (Figs. 22 and 23).

### 6. Thyroiditis

**Figure 24:**
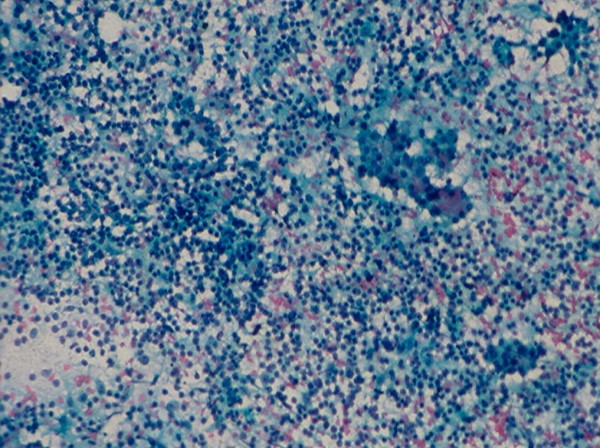
Hashimoto thyroiditis showing in FNA numerous lymphoid cells admixed with a sheet of follicular epithelial cells (Papanicolaou stain, × 100).

**Figure 25:**
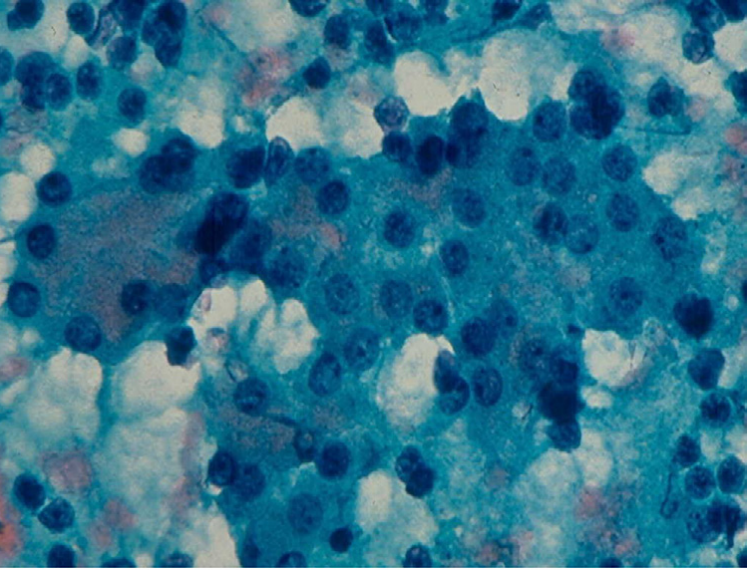
A sheet of follicular epithelial cells with oncocytic change admixed with benign lymphoid cells seen in FNA of a Hashimoto thyroiditis (Papanicolaou stain, × 400).

**Figure 26:**
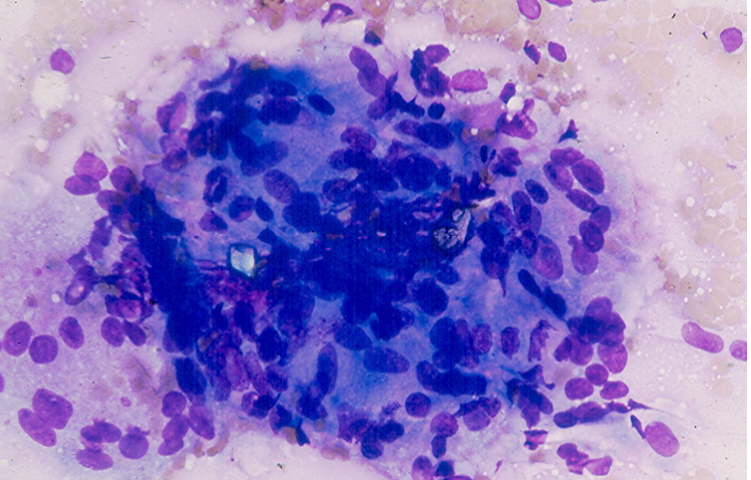
A syncytial cluster of epithelioid cells with carrot-shaped nuclei seen in FNA of a subacute thyroiditis (Diff-Quik stain, × 400).

**Figure 27:**
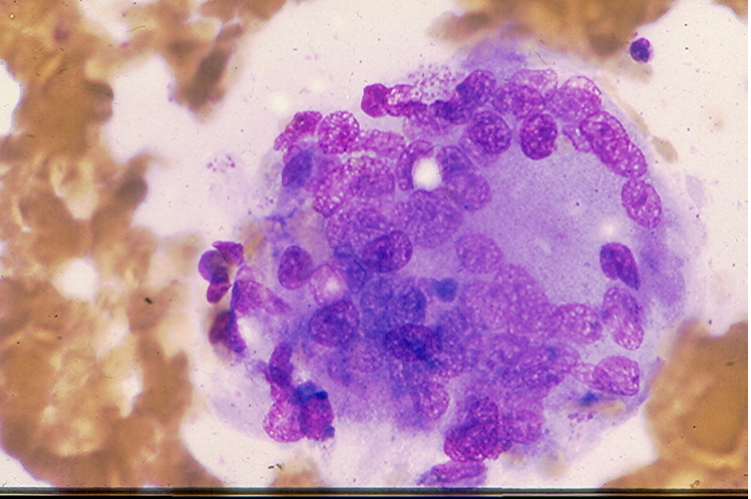
A large multinucleate giant cell present in FNA of a subacute thyroiditis (Diff-Quik stain, × 400).

*Hashimoto thyroiditis *and *subacute thyroiditis *commonly have fairly distinctive clinical findings. Rarely, these lesions may present as a nodular lesion mimicking a thyroid neoplasm. Hashimoto thyroiditis is characterized by the presence of numerous benign lymphoid cells admixed with benign follicular cells and Hurthle cells, (Figs. 24 and 25). A subacute thyroiditis may yield clustered epithelioid cells, scattered lymphocytes and a few multinucleated giant cells containing up to one hundred nuclei [13,22-25,37] (Figs. 26 and 27). It should be born in mind that Hashimoto thyroiditis may harbor hyperplastic follicular and Hurthle cell nodules, and these two nodules are cytologically indistinguishable from a cellular follicular neoplasm and a Hurthle cell neoplasm, respectively [37,38]. Surgical excision of these lesions is usually required for histologic confirmation.

### 7. Other Lesions

*Graves disease *may rarely present as a nodular thyroid lesion [55]. It yields non specific cytologic findings [13].

*Metastatic cancers *to the thyroid are common in patients with advanced cancers arising from other body sites [25]. However, metastatic cancer to the thyroid gland presenting as a palpable TN is uncommon. For unknown reasons, renal cell carcinoma is the most common metastatic neoplasm to the thyroid, and cases of clinically occult renal cell carcinoma presenting initially as a large thyroid mass have been documented [25]. Cytodiagnosis of metastatic cancer to the thyroid is relatively straightforward as metastatic cancer usually displays a cytologic pattern distinctive from those of a primary thyroid carcinoma [25]. However, a cytological differential diagnosis between a metastatic renal cell carcinoma of clear cell type and a primary thyroid carcinoma with clear cell change may be difficult, and immunocytochemical staining of aspirated tumor cells with thyroglobulin antibody will be helpful to identify the aforementioned primary thyroid cancer.

### 8. Non-Diagnostic Category

The lesions in this category are highly diversified and may be any lesions listed in the above seven categories. In this category the FNA yields non-diagnostic or inadequate cellular materials. In one study, cystic thyroid lesions yielded non-diagnostic cell samples at initial FNA in about 50% of cases [12]. In the Mayo Clinic experience, repeating the FNA in the cases with initial non-diagnostic needle aspirates revealed diagnostic material in 30 to 80% of cases [12,15]. Other investigators found that thyroid re-FNA was of limited value [59]. If the re-aspiration is still non-diagnostic, ultrasound-guided FNA should be performed. Ultrasound-guided FNAs yield adequate cytologic materials in about 91% of cases [27-36]. Patients with no specific risk factors for thyroid malignancy and a non-diagnostic FNA who refuse a re-biopsy may be managed conservatively. While patients in the high-risk group should have their TNs removed for histologic study, an increase in nodule volume alone is not a reliable predictor of malignancy, as most solid and benign TNs grow in size [57].

## Diagnostic Accuracy and Errors

In a review of seven large series totaling 18,183 thyroid FNAs, Gharib and Goellner found that the biopsy technique had a sensitivity rate varying from 65 to 98% (mean 83%), and that its specificity rate varied from 72 to 100% (mean 92%) [15]. The false-negative rate varied from 1 to 11.5% (mean, 5.2%), and the false-positive rate varied from 0 to 7.7% (mean, 2.9%) [15]. The overall cytodiagnostic accuracy rate of thyroid FNA approached 95% according to some reported series [13].

## Adjunctive Diagnostic Value of Ancillary Techniques

Ultrafast Papanicolaou stain selectively swells the nuclei of papillary thyroid carcinoma, making their nuclear grooves disappear and making the swollen nuclei look like "watery grapes", while this staining method has no effect on nuclei of a follicular adenoma [21]. This artifactual change is due to the disorganization of nuclear lamins and permits a confident distinction between a follicular adenoma and a follicular variant papillary carcinoma [21]. Immunostaining with thyroid peroxidase antibody has been reported to be of value in distinguishing these two lesions, as malignant and benign follicular cells commonly stain negatively and positively with this antibody, respectively [58].

Ploidy determination has no value in distinguishing a follicular adenoma from a follicular carcinoma [59-62] and immunostaining for p53, Ki-67 and Bcl-2 has no value in separating benign from malignant Hurthle cell tumors [63].

Genetics-molecular studies have been extensively carried out on tissue samples of different types of thyroid neoplasm since the past decade [64]. However, only a few genetics-molecular studies on thyroid cells obtained by FNA have been recently published. Human telomerase reverse transcriptase (hTERT) gene expression, using reverse transcriptase-polymerase chain reaction, has been identified as a promising diagnostic marker in distinguishing benign from malignant tumors in materials obtained by FNA. It was found that 90 and 92.8% of thyroid carcinomas were positive for hTERT while 35 and 61.5% of benign thyroid nodules were positive for hTERT, respectively [65,66]. Among the thyroid tumors with positive hTERT, there were eight of eight papillary, two of two Hurthle cell and three of four follicular carcinomas [65]. BRAF point mutation and RET/thyroid PC rearrangements were found in 38% of thyroid PCs and refined the diagnosis of thyroid PC in five of fifteen cell samples that were considered either indeterminate or insufficient by cytology. No mutation was found in FNAs of follicular adenomas and non-toxic nodular goiters [67]. These molecular markers were of adjunctive diagnostic value when the FNA diagnosis of TN was equivocal [65-67].

Powerful molecular techniques including microarray analysis and molecular profiling may have a significant role in the future evaluation of TNs, while providing impetus for further insight into the molecular pathogenesis of both benign and malignant TNs [68-71]. Moreover, such techniques may allow deeper insight into both loss and gain of function of unidentified genes by examining panels of genes rather than one or a limited number of potential gene candidates. By analysis of cancer gene profiles for a cohort of 62 thyroid samples, Finley et al [68] were able to distinguish between benign and malignant thyroid tumors. They reported a sensitivity of 91.7% and specificity of 96.2% for the detection of thyroid carcinomas of various types, including thyroid PC and its follicular variant and follicular carcinoma [68]. Distinction of benign and malignant thyroid tumors and molecular classification of follicular thyroid tumors by gene profiling suggests that these powerful techniques may have significant diagnostic potential when used with FNA cytology [69,70]. Molecular profiling may also permit the distinction between primary and metastatic malignancies when dealing with multiple suspicious nodules at various sites. Using material retrieved by FNA, Schoedel et al [71], compared loss of heterozygosity (LOH) patterns and demonstrated genetic kinship of multifocal carcinomas in the thyroid and a separate nodule in the lung, supporting a diagnosis of metastatic thyroid carcinoma to the lung rather than an independent lung neoplasm.

At present, techniques such as microarray analysis are limited by the amount of RNA that can be retrieved from a sample, thereby often limiting analysis to surgically resected samples. However, refinement of these techniques may make them applicable to FNA, with extraction of RNA from a cell block from which molecular analysis of FNA material may have significant diagnostic benefit.

## Acknowledgements

Co-editors of Cytojournal Vinod B. Shidham, MD, FRCpath, FIAC, and Barbara F. Atkinson, MD thank the academic editor: Zubair W. Baloch, MD, PhD.

Hospital of the University of Pennsylvania Pathology, 6 Founders, 3400 Spuce St, Philadelphia, PA 19104, USA Email: baloch@mail.med.upenn.edu.
